# Radial nerve palsy associated with closed humeral shaft fractures: a systematic review of 1758 patients

**DOI:** 10.1007/s00402-020-03446-y

**Published:** 2020-04-13

**Authors:** Laurent A. M. Hendrickx, Nick F. J. Hilgersom, Hassanin Alkaduhimi, Job N. Doornberg, Michel P. J. van den Bekerom

**Affiliations:** 1 Department of Orthopaedic Surgery, Amsterdam University Medical Centre, University of Amsterdam, Amsterdam Movement Sciences, Amsterdam, The Netherlands; 2 Department of Orthopaedic and Trauma Surgery, Flinders Medical Centre, Flinders University, Adelaide, 5042 Australia; 3 Department of Orthopaedic Surgery, Onze Lieve Vrouwe Gasthuis, 1091 Amsterdam, The Netherlands

**Keywords:** Humerus, Radial nerve, Humeral shaft fracture, Fracture, Nerve palsy, Nerve injury

## Abstract

**Background and purpose:**

Humeral shaft fractures are often associated with radial nerve palsy (RNP) (8–16%). The primary aim of this systematic review was to assess the incidence of primary and secondary RNP in closed humeral shaft fractures. The secondary aim was to compare the recovery rate of primary RNP and the incidence of secondary RNP between operative and non-operative treatment.

**Methods:**

A systematic literature search was performed in ‘Trip Database’, ‘Embase’ and ‘PubMed’ to identify original studies reporting on RNP in closed humeral shaft fractures. The Coleman Methodology Score was used to grade the quality of the studies. The incidence and recovery of RNP, fracture characteristics and treatment characteristics were extracted. Chi-square and Fisher exact tests were used to compare operative versus non-operative treatment.

**Results:**

Forty studies reporting on 1758 patients with closed humeral shaft fractures were included. The incidence of primary RNP was 10%. There was no difference in the recovery rate of primary RNP when comparing operative treatment with radial nerve exploration (98%) versus non-operative treatment (91%) (*p* = 0.29). The incidence of secondary RNP after operative and non-operative treatment was 4% and 0.4%, respectively (*p* < 0.01).

**Interpretation:**

One-in-ten patients with a closed humeral shaft fracture has an associated primary RNP, of which > 90% recovers without the need of (re-)intervention. No beneficial effect of early exploration on the recovery of primary RNP could be demonstrated when comparing patients managed non-operatively with those explored early. Patients managed operatively for closed humeral shaft fractures have a higher risk of developing secondary RNP.

**Level of evidence:**

Level IV; Systematic Review.

**Electronic supplementary material:**

The online version of this article (10.1007/s00402-020-03446-y) contains supplementary material, which is available to authorized users.

## Introduction

Humeral shaft fractures account for 1–3% of all fractures [1]. The incidence, 14 per 100,000 per year [1, 2], has increased over 100% over the past 25 years [3]. Due to its course around the humerus, the radial nerve is prone to injury in these fractures: radial nerve palsy (RNP) has been reported in 8–16% of humeral shaft fractures [1, 4, 5].

Radial nerve palsy can either be primary or secondary. Primary RNP occurs at the onset of the fracture and may be caused by compression within the fracture site, transection by fracture fragments, or local pressure by swollen tissue. Secondary, or iatrogenic, RNP develops during the course of treatment. Secondary RNPs account for 10–20% of all RNPs associated with humeral shaft fractures [6].

Though frequently seen, it remains unclear what the best treatment strategy for RNP associated with humeral shaft fractures is. Some recommend conservative management based on the experience that RNP often resolves spontaneously. A primary RNP following a closed humeral shaft fracture has been reported to recover in > 70% [4, 6, 7]. Others suggest surgical management. They state early exploration can aid in diagnosing the type of RNP (e.g., neuropraxia) and may be associated with a reduced risk of nerve entrapment by scar tissue or exuberant callus [4, 6]**.** Early exploration also gives the opportunity to repair a lacerated nerve at an early stage, which may result in better outcome [6].

The most recent review investigating RNP in humeral shaft fractures dates to 2013 [5]; however, over the last 6 years 20 studies have been added to potentially contribute to our understanding [8–27]. Therefore, the primary aim of this study was to systematically review all contemporary evidence to assess the incidence and recovery rate of primary and secondary RNP in closed humeral shaft fractures. The secondary aim was to compare the recovery rate of primary RNP and the incidence of secondary RNP between operative and non-operative treatment.

## Materials and methods

### Protocol

This study adhered to the Preferred Reporting Items for Systematic Reviews and Meta-Analyses (PRISMA) guidelines [28]. A study protocol was created prior to commencement of the study. The protocol was not registered.

### Selection criteria

All original studies that assessed the outcome of operative or non-operative treatment of traumatic humeral shaft fractures were included. Studies had to report on the presence or absence of primary or secondary RNP to be included. Only studies including at least ten adult (i.e., ≥ 18 years) patients with closed, non-pathological humeral shaft fractures were included. Studies that reported solely on patients with RNP were excluded to avoid selection bias. The inclusion and exclusion criteria are summarized in Table 1.

**Table 1:** Inclusion and exclusion criteria

Inclusion criteria	Exclusion criteria
Studies including ≥ 10 adult (≥ 18 years) patients with closed, traumatic humeral shaft fractures	Studies not reporting on the presence or absence of RNP
Cohort studies, case series, RCTs	Studies not reporting on the length of follow-up
	Open fracture, pathological fractures, non-union

### Literature search strategy

In collaboration with a clinical librarian ‘Trip database’, ‘PubMed’ and ‘Embase’ were searched to gather all available evidence on primary and secondary RNP associated with closed humeral shaft fractures. Details of the searches are displayed in Table 2. Searches were limited to English, German and Dutch papers, published since 1990. Searches were updated until 11 June 2019. Reference lists of included studies were manually searched to assure no studies meeting inclusion criteria were missed.

**Table 2:** Literature search PubMed, Embase and Trip

Database	Search terms
PubMed	("Humerus"[Mesh] OR Humer*) *AND* (("Radial Nerve"[Mesh]) OR (Radial AND (nerve OR nerves OR nervus OR nervous OR neuropathy OR palsy OR palsies OR paralys*))) *AND* (("Fractures, Bone"[Mesh]) OR Fractur*)
Embase and Trip	humer* *AND* (radial AND (nerve OR nerves OR nervus OR nervous OR neuropathy OR palsy OR palsies OR paralys*)) *AND* fractur*

### Screening for eligibility

Two authors (LH and NH) independently screened the title, abstracts and full texts of the studies for eligibility based on predetermined inclusion- and exclusion criteria. Disagreement was resolved by discussion. If no consensus could be reached, a senior author (MB) gave the final verdict.

### Assessment of quality

Two authors (LH and NH) independently assessed the quality of the studies using the ‘Coleman Methodology Score’ [29]. The ‘Coleman Methodology Score’ was adjusted to make it more suitable for the studies in the systematic review (Appendix A). The total score corresponded to either a poor (0–49), fair (50–69), good (70–84), or excellent (85–100) quality of the study. Mismatches in assigned scores were discussed by two authors (LH and NH). If no agreement could be reached, a senior author made the final decision (MB).

### Data extraction

The following data were extracted from the included studies by one author (LH) and validated by a second author (NH): study characteristics, study design, patient characteristics, fracture characteristics, primary RNP, secondary RNP, recovery of RNP, time to recovery of RNP, type of treatment and type of approach used. Surgical approaches were categorized into three groups: (1) anterolateral (i.e. minimally invasive anterior, open anterolateral and open extended deltopectoral); (2) posterior (i.e., open posterior and minimally invasive posterior); and (3) lateral (i.e., lateral open). A more detailed description of the extracted data is displayed in the appendix (Appendix B).

### Statistical analysis

Results were summarized as absolute numbers with percentages. Patients with primary radial neve palsy were not included in the analysis of the incidence of secondary RNP. Fisher’s exact tests and Chi-square tests were used to compare the recovery rate of primary RNP and the incidence of secondary RNP in operative versus non-operative treatment of closed humeral shaft fractures.

## Results

### Study selection

**Fig. 1:**
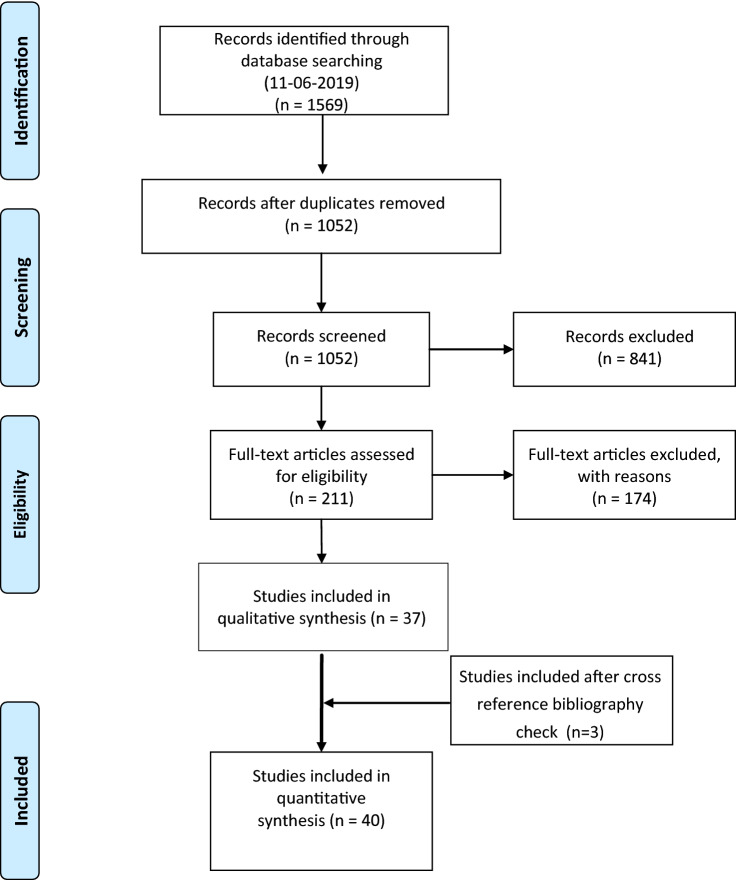
Flow chart of study selection process

1052 unique studies were identified, of which 37 met the inclusion criteria. Three additional studies were identified through cross-referencing, resulting in the final inclusion of 40 studies [8–27, 30–49]. The flowchart of the selection process is displayed in Fig. 1.

### Critical appraisal

Median Coleman score was 56 (range 22–91), indicating a fair overall quality of included studies. 12 studies were ranked poor [9–11, 21, 34–39, 47, 49], 24 studies were ranked fair [8, 12, 14–18, 20, 22–27, 30–33, 40, 41, 43, 45, 46, 48], 3 studies were ranked good [13, 42, 44] and 1 study was ranked excellent [19].

### Study characteristics

The 40 included studies described 1758 patients with closed humeral shaft fractures. Study sizes ranged from 10 to 212 patients. Follow-up varied from 3 to 75 months.

There were four randomized controlled trials (RCTs) [13, 19, 41, 42], six prospective studies [8, 12, 43–46] and 30 retrospective studies [9–11, 14–18, 20–27, 30–40, 47–49]. One study was written in German [49], and the remaining studies were written in English. 24 studies were conducted in Asia [11–13, 15–18, 20, 22–25, 27, 30, 31, 33, 36–38, 40–42, 44, 46, 48], six in Europe [21, 32, 39, 45, 47, 49], five in South-America [14, 19, 26, 34, 43], three in North-America [9, 10, 35] and one in Africa [8].

Two studies reported solely on the effect of non-operative management [20, 45], five studies reported on the effect of operative and non-operative management [9, 10, 19, 35, 47] and the remaining studies reported on the effect of one or more surgical interventions [8, 11–18, 21–27, 30–34, 36–46, 48, 49]. Study characteristics are displayed in Appendix C.

### Incidence primary RNP

22 studies reported on primary RNP [9–12, 14, 16, 18, 20, 22, 23, 26, 33–35, 37–40, 43, 45, 47, 49]. These studies consisted of 922 patients of which 88 presented with primary RNP (10%) (Table 3).

**Table 3:** Incidence and recovery rate of primary radial nerve palsy in closed humeral shaft fractures

	Overall *N* = 922^a^	Non-operative *N* = 240	Operative *N* = 682	*p *value
Primary RNP, *n* (%)	88 (10%)	23 (10%)	65 (10%)	
Recovery without re-intervention, *n* (%)	83 (94%)	21 (91%)	62 (95%)	0.60

^a^18 studies (*n* = 836) excluded patients with primary radial nerve palsy and are not included in this analysis

Primary RNP was reported in 0 out of 17 proximal 1/3rd, 5 out of 67 middle 1/3rd and 19 out of 152 (13%) distal 1/3rd humeral shaft fractures (Table 4).

**Table 4:** Fracture characteristics and primary radial nerve palsy

	*N*	Primary RNP
Fracture location		
Proximal 1/3	17	0
Middle 1/3	67	5
Distal 1/3	152	19 (13%)
Fracture type		
Spiral	62	10
Oblique	25	3
Transverse	47	2
Comminuted	97	5

Primary RNP was seen in 10 out of 62 spiral, 3 out of 25 oblique, 2 out of 47 transverse and 5 out of 97 comminuted humeral shaft fractures.

### Recovery of primary RNP

The overall recovery rate of primary RNP was 94% (Table 3). The longest time to full spontaneous recovery of primary RNP reported was 12 months [10].

The recovery rate of primary RNP in non-operatively managed humeral shaft fractures was 91%. In 21 out of 23 cases the radial nerve showed full recovery without reintervention. Definitive management of the patients without recovery of primary RNP was not reported [10, 20].

The recovery rate of primary RNP in operatively treated humeral shaft fractures was 95%. 62 out of 65 primary RNPs showed full recovery without reintervention. In 41 of these 65, the radial nerve was explored during surgery, of which 40 (98%) showed full recovery. One of three patients not recovering from primary RNP after initial operative treatment was managed with a tendon transfer after 6-months [35], another patient underwent radial nerve exploration after 3 months during which the radial nerve was released from callus [37]. In the third patient, definitive management was not reported [10].

There was no significant difference in the recovery rate of primary RNP for the initial operative or non-operative treatment of closed humeral shaft fractures (*p* = 0.60), nor was there a difference when operative treatment with nerve exploration was compared to non-operative treatment (*p* = 0.29).

### Incidence of secondary RNP

All studies reported on secondary RNP. In 57 out of 1670 (3%) patients, treatment was complicated by secondary RNP (Table 5) The incidence of secondary RNP in non-operatively treated patients was 0.4%, while the incidence of secondary RNP in operatively treated patients was 4%. This difference was statistically significant (*p* < 0.01).

**Table 5:** Incidence and recovery rate of secondary radial nerve palsy in closed humeral shaft fractures

	Overall *N* = 1670^a^	Non-operative *N* = 269^a^	Operative *N* = 1401^a^	*p *value
Secondary RNP, *n* (%)	57 (4%)	1 (0.4%)	56 (4%)	< 0.01*
Recovery without reintervention, *n* (%)	51/54 (94%)^b^	0 (0%)	51/53 (96%)^b^	

^a^Patients with primary RNP were not included in this analysis

^b^Arora et al. did not describe the recovery of three secondary RNPs. These were not included in the recovery rate

*Chi-square test was significant

### Fixation technique and secondary radial nerve palsy

11 studies [11–13, 15, 21, 30, 33, 37, 38, 42, 49] assessed the outcome of intramedullary nailing, describing a total of 385 patients. In seven patients (2%), treatment was complicated by secondary RNP (Table 6).

**Table 6:** Surgical technique and incidence of secondary radial nerve palsy

	Total patients treated *N*^a^	Secondary RNP *n* (%)
Fixation technique		
IMN	385	7 (2%)
Conventional plating	553	36 (7%)
MIPO	436	12 (3%)
Surgical approach		
Anterolateral approach	467	14 (3%)
Posterior approach	356	17 (5%)
Lateral approach	46	0

^a^Patients with primary RNP were not included in this analysis

17 studies [8, 14, 17, 19, 24, 26, 27, 30–32, 34, 36, 39, 43, 44, 46, 48] reported on the effect of minimally invasive plate osteosynthesis, consisting of 436 patients. In 12 patients (3%), treatment was complicated by secondary RNP.

13 studies [13, 16–18, 22, 23, 25, 33, 35, 40–42, 48] reported on 553 patients treated with conventional plating. In 36 patients (7%) treatment was complicated by secondary RNP.

### Surgical approach and secondary RNP

In 14 out of 467 patients (3%) in whom an anterolateral approach was used [17–19, 22, 24, 26, 27, 30–32, 34, 36, 39, 43, 44, 46, 48] and in 17 out of 356 (5%) patients in whom a posterior approach was used [8, 14, 23, 25, 40, 41], treatment was complicated by secondary RNP (Table 6). In none of the 46 patients in whom a lateral approach was used secondary RNP occurred [16, 25].

### Recovery from secondary RNP

21 studies [8, 10–15, 17, 19, 21–23, 25, 27, 33, 35, 36, 40, 42, 44, 48], with a weighted mean follow-up of 19 months, described the recovery from secondary RNP. 51 out of 54 patients with secondary RNPs recovered without reintervention (94%) (Table 5). The longest time to spontaneous recovery of secondary RNP reported was 12 months [48]. In three patients, secondary RNP did not resolve without reintervention [35, 44]. One patient was initially managed non-operatively and went on to conventional plating 2 months later because of unresolved secondary RNP and malalignment. The radial nerve was found to be intact and recovered after surgery [35]. The other two patients were managed operatively. One developed secondary RNP immediately after minimally invasive plate osteosynthesis. This patient was re-operated and underwent surgical exploration with plate reapplication after which the RNP resolved within 48 h [44]. The other patient was initially managed operatively with conventional plating. In this patient, the radial nerve showed no recovery at final follow-up 3 months after surgery [35].

## Discussion

One-in-ten patients with a closed humeral shaft fracture has an associated primary RNP, of which more than 90% recovers without the need of any (re-)intervention. No beneficial effect of early exploration on the recovery of primary RNP could be demonstrated when comparing patients managed non-operatively with those explored early. Moreover, patients managed operatively for a closed humeral shaft fracture had a significantly higher risk of developing secondary RNP.

The previously described findings should be appreciated with several limitations in mind. Firstly, the overall methodological quality of the studies was fair and included only three RCTs, while it furthermore consisted of observational studies. Although previous studies have also used this methodology of combining both RCT’s and observational trials [50, 51], it is important to note that the risk of bias is substantially larger in observational studies. Secondly, the heterogeneity of the used interventions between included studies was high. Due to these limitations (i.e., limited methodological quality and heterogeneity), a proper meta-analysis could not be conducted, and results of this study should therefore not be interpreted as such. However, we do believe that this study may provide sufficient evidence to compare non-operative versus operative treatment, as it is difficult to conduct a study of higher methodological quality such as an RCT or meta-analysis. The former is difficult to conduct due to the low incidence of RNP, while the latter is not possible due to the absence of high-quality studies. The strengths of this systematic review include: (1) a comprehensive search of the literature in collaboration with a clinical librarian; (2) selection of studies and grading of the evidence by two authors independently; and (3) large number of studies included resulting in the largest series of closed humeral shaft fractures to date. Given these strengths, we believe that the reported incidence for primary and secondary RNP are accurate estimates of the true incidence in patients with closed humeral shaft fractures.

The incidence of primary RNP associated with closed humeral shaft fractures observed in this systematic review was 10%. Previous studies have reported on incidences ranging from 8 to 12% [1, 4, 52]. However, these studies included patients with open fractures [1, 4, 52], pathological fractures [1], or were based on smaller numbers [1, 52].

In the current study, the radial nerve appeared to be particularly at risk in middle (7%) and distal third (13%) humeral shaft fractures as well as in spiral-type fractures (16%). This is in line with previous studies reporting increased risks of primary RNP for these specific type of fractures [1, 4, 5, 53].

The overall recovery rate of primary RNP was 94%. No significant difference in the recovery rate could be demonstrated when comparing non-operative treatment versus operative treatment with nerve exploration. We therefore support earlier recommendations [4, 54] to manage patients with a closed humeral shaft fracture and an associated RNP non-operatively if the fracture allows it. This avoids the risk of potential operative complications and does not affect the recovery of the radial nerve adversely, while high union rates have been demonstrated with non-operative management in closed humeral shaft fractures [55].

The current study demonstrated a significantly lower incidence of secondary RNP in patients who were treated non-operatively for closed humeral shaft fractures (0.4%) compared to patients who received operative treatment (4%). Other large series assessing secondary RNP after operative treatment in open and closed humeral shaft fractures report incidences ranging from 6 to 7% [24, 53, 56]. This may advocate non-operative treatment in closed humeral shaft fractures if the fracture is expected to heal well with non-operative treatment.

Although the use of ultrasound as a diagnostic modality in patients with RNP after humeral shaft fractures has become more common over the years, it is currently not part of the standard diagnostic workup. Bodner and colleagues were the first to describe the use of ultrasound in patients with RNP after humerus shaft fractures [57, 58]. In a prospective study including 11 patients, they correctly diagnosed the type of RNP based on their pre-operative ultrasound findings in all five patients who underwent nerve exploration [58]. A more recent study further highlights the potential of ultrasound in diagnosing RNP in humeral shaft fractures: in 11 out 12 patients undergoing radial nerve exploration, the pre-operative ultrasound findings were confirmed intra-operatively [59]. It is important to note that for both studies, it cannot be confirmed whether ultrasound findings in patients not undergoing nerve exploration were also correct [58, 59]. This limitation was overcome in a cadaveric study by Cartwright et al., demonstrating the ability of ultrasound to diagnose nerve transection with a sensitivity and specificity of 89% and 95%, respectively [60]. Given its non-invasive nature and its supposed accurate ability to diagnose and differentiate between various types of nerve injury, further research on the use of ultrasound in the diagnostic workup of RNP in humeral shaft fractures is merited.

In conclusion, this study demonstrated that one-in-ten patients with a closed humeral shaft fracture has an associated primary RNP. No significant difference in the recovery rate of primary RNP could be demonstrated when comparing groups which were initially managed non-operatively with those explored early. This suggests that that non-operative treatment does not affect the extent of nerve recovery adversely. Non-operative treatment of closed humeral shaft fractures is furthermore associated with a significantly lower risk of secondary RNP.

## Electronic supplementary material

Below is the link to the electronic supplementary material.

Supplementary file1 (PDF 105 kb)

Supplementary file2 (PDF 96 kb)

Supplementary file3 (PDF 124 kb)

## References

[1] Ekholm R, Adami J, Tidermark J (2006). Fractures of the shaft of the humerus. An epidemiological study of 401 fractures. J Bone Joint Surg Br.

[2] Bergdahl C, Ekholm C, Wennergren D (2016). Epidemiology and patho-anatomical pattern of 2,011 humeral fractures: data from the Swedish Fracture Register. BMC Musculoskelet Disord.

[3] Mahabier KC, Hartog DD, Van Veldhuizen J (2015). Trends in incidence rate, health care consumption, and costs for patients admitted with a humeral fracture in The Netherlands between 1986 and 2012. Injury.

[4] Shao YC, Harwood P, Grotz MRW (2005). Radial nerve palsy associated with fractures of the shaft of the humerus: a systematic review. J Bone Joint Surg Br.

[5] Li Y, Ning G, Wu Q (2013). Review of literature of radial nerve injuries associated with humeral fractures-an integrated management strategy. PLoS ONE.

[6] DeFranco MJ, Lawton JN (2006). Radial nerve injuries associated with humeral fractures. J Hand Surg Am.

[7] Chang G, Ilyas AM (2018). Radial nerve palsy after humeral shaft fractures: the case for early exploration and a new classification to guide treatment and prognosis. Hand Clin.

[8] Balam KM, Zahrany AS (2014). Posterior percutaneous plating of the humerus. Eur J Orthop Surg Traumatol.

[9] Belayneh R, Lott A, Haglin J (2019). Final outcomes of radial nerve palsy associated with humeral shaft fracture and nonunion. J Orthop Traumatol.

[10] Dielwart C, Harmer L, Thompson J (2017). Management of closed diaphyseal humerus fractures in patients with injury severity score ≥17. J Orthop Trauma.

[11] Duygun F, Aldemir C (2017). Is locked compressive intramedullary nailing for adult humerus shaft fractures advantageous?. Eklem Hastalik Cerrahisi.

[12] Ebrahimpour A, Najafi A, Manafi Raci A (2015). Outcome assessment of operative treatment of humeral shaft fractures by antegrade unreamed humeral nailing (UHN). Indian J Surg.

[13] Fan Y, Li Y-W, Zhang H-B (2015). Management of humeral shaft fractures with intramedullary interlocking nail versus locking compression plate. Orthopedics.

[14] Gallucci GL, Boretto JG, Alfie VA (2015). Posterior minimally invasive plate osteosynthesis (MIPO) of distal third humeral shaft fractures with segmental isolation of the radial nerve. Chir Main.

[15] Han KJ, Lee DH, Bang JY (2017). Do Cerclage cables delay the time to bone union in patients with an unstable humeral shaft fracture treated with intramedullary nails?. Yonsei Med J.

[16] Kharbanda Y, Tanwar YS, Srivastava V (2017). Retrospective analysis of extra-articular distal humerus shaft fractures treated with the use of pre-contoured lateral column metaphyseal LCP by triceps-sparing posterolateral approach. Strategies Trauma Limb Reconstr.

[17] Ko S-H, Cha J-R, Lee CC (2017). Minimally invasive plate osteosynthesis using a screw compression method for treatment of humeral shaft fractures. Clin Orthop Surg.

[18] Lee HM, Kim YS, Kang S (2018). Modified anterolateral approach for internal fixation of Holstein-Lewis humeral shaft fractures. J Orthop Sci.

[19] Matsunaga FT, Tamaoki MJS, Matsumoto MH (2017). Minimally invasive osteosynthesis with a bridge plate versus a functional brace for humeral shaft fractures: a randomized controlled trial. J Bone Joint Surg Am.

[20] Pal JN, Biswas P, Roy A (2015). Outcome of humeral shaft fractures treated by functional cast brace. Indian J Orthop.

[21] Salvador J, Amhaz-Escanlar S, Castillón P (2018). Cerclage wiring and intramedullary nailing, a helpful and safe option specially in proximal fractures. A multicentric study. Injury.

[22] Seo J-B, Heo K, Yang J-H, Yoo J-S (2019). Clinical outcomes of dual 3.5-mm locking compression plate fixation for humeral shaft fractures: Comparison with single 4.5-mm locking compression plate fixation. J Orthop Surg.

[23] Singh AK, Narsaria N, Seth RR, Garg S (2014). Plate osteosynthesis of fractures of the shaft of the humerus: comparison of limited contact dynamic compression plates and locking compression plates. J Orthop Traumatol.

[24] Wang Q, Hu J, Guan J (2018). Proximal third humeral shaft fractures fixed with long helical PHILOS plates in elderly patients: benefit of pre-contouring plates on a 3D-printed model-a retrospective study. J Orthop Surg Res.

[25] Yin P, Zhang L, Mao Z (2014). Comparison of lateral and posterior surgical approach in management of extra-articular distal humeral shaft fractures. Injury.

[26] Zogaib RK, Morgan S, Belangero PS (2014). Minimal invasive ostheosintesis for treatment of diaphiseal transverse humeral shaft fractures. Acta Ortop Bras.

[27] Shen L, Qin H, An Z (2013). Internal fixation of humeral shaft fractures using minimally invasive plating: comparative study of two implants. Eur J Orthop Surg Traumatol.

[28] Moher D, Liberati A, Tetzlaff J (2009). Preferred reporting items for systematic reviews and meta-analyses: the PRISMA statement. BMJ.

[29] Coleman BD, Khan KM, Maffulli N (2000). Studies of surgical outcome after patellar tendinopathy: clinical significance of methodological deficiencies and guidelines for future studies. Scand J Med Sci Sports.

[30] An Z, He X, Jiang C, Zhang C (2012). Treatment of middle third humeral shaft fractures: minimal invasive plate osteosynthesis versus expandable nailing. Eur J Orthop Surg Traumatol.

[31] Apivatthakakul T, Phornphutkul C, Laohapoonrungsee A, Sirirungruangsarn Y (2009). Less invasive plate osteosynthesis in humeral shaft fractures. Oper Orthop Traumatol.

[32] Brunner A, Thormann S, Babst R (2012). Minimally invasive percutaneous plating of proximal humeral shaft fractures with the Proximal Humerus Internal Locking System (PHILOS). J Shoulder Elbow Surg.

[33] Chao T-C, Chou W-Y, Chung J-C, Hsu C-J (2005). Humeral shaft fractures treated by dynamic compression plates, ender nails and interlocking nails. Int Orthop.

[34] Concha JM, Sandoval A, Streubel PN (2010). Minimally invasive plate osteosynthesis for humeral shaft fractures: are results reproducible?. Int Orthop.

[35] Jawa A, McCarty P, Doornberg J (2006). Extra-articular distal-third diaphyseal fractures of the humerus. A comparison of functional bracing and plate fixation. J Bone Joint Surg Am.

[36] Lau TW, Leung F, Chan CF, Chow SP (2007). Minimally invasive plate osteosynthesis in the treatment of proximal humeral fracture. Int Orthop.

[37] Liebergall M, Jaber S, Laster M (1997). Ender nailing of acute humeral shaft fractures in multiple injuries. Injury.

[38] Lin J, Hou S-M (2003). Locked nailing of severely comminuted or segmental humeral fractures. Clin Orthop Relat Res.

[39] Spagnolo R, Pace F, Bonalumi M (2010). Minimally invasive plating osteosynthesis technique applied to humeral shaft fractures: the lateral approach. Eur J Orthop Surg Traumatol.

[40] Yang Q, Wang F, Wang Q (2012). Surgical treatment of adult extra-articular distal humeral diaphyseal fractures using an oblique metaphyseal locking compression plate via a posterior approach. Med Princ Pract.

[41] Arora S, Goel N, Cheema GS (2011). A method to localize the radial nerve using the “apex of triceps aponeurosis” as a landmark. Clin Orthop Relat Res.

[42] Li Y, Wang C, Wang M (2011). Postoperative malrotation of humeral shaft fracture after plating compared with intramedullary nailing. J Shoulder Elbow Surg.

[43] Livani B, Belangero WD (2004). Bridging plate osteosynthesis of humeral shaft fractures. Injury.

[44] Malhan S, Thomas S, Srivastav S (2012). Minimally invasive plate osteosynthesis using a locking compression plate for diaphyseal humeral fractures. J Orthop Surg.

[45] Pehlivan O (2002). Functional treatment of the distal third humeral shaft fractures. Arch Orthop Trauma Surg.

[46] Zhiquan A, Bingfang Z, Yeming W (2007). Minimally invasive plating osteosynthesis (MIPO) of middle and distal third humeral shaft fractures. J Orthop Trauma.

[47] Ekholm R, Ponzer S, Törnkvist H (2008). The Holstein-Lewis humeral shaft fracture: aspects of radial nerve injury, primary treatment, and outcome. J Orthop Trauma.

[48] An Z, Zeng B, He X (2010). Plating osteosynthesis of mid-distal humeral shaft fractures: minimally invasive versus conventional open reduction technique. Int Orthop.

[49] Habernek H, Schmid L, Orthner E (1992). Initial experiences with the humerus interlocking nail. Unfallchirurgie.

[50] Veltman ES, Doornberg JN, Eygendaal D, van den Bekerom MPJ (2015). Static progressive versus dynamic splinting for posttraumatic elbow stiffness: a systematic review of 232 patients. Arch Orthop Trauma Surg.

[51] Iliaens J, Metsemakers W-J, Coppens S (2019). Regional anaesthesia for surgical repair of proximal humerus fractures: a systematic review and critical appraisal. Arch Orthop Trauma Surg.

[52] Tsai C-H, Fong Y-C, Chen Y-H (2009). The epidemiology of traumatic humeral shaft fractures in Taiwan. Int Orthop.

[53] Claessen FMAP, Peters RM, Verbeek DO (2015). Factors associated with radial nerve palsy after operative treatment of diaphyseal humeral shaft fractures. J Shoulder Elbow Surg.

[54] Liu G-Y, Zhang C-Y, Wu H-W (2012). Comparison of initial nonoperative and operative management of radial nerve palsy associated with acute humeral shaft fractures. Orthopedics.

[55] Sarmiento A, Zagorski JB, Zych GA (2000). Functional bracing for the treatment of fractures of the humeral diaphysis. J Bone Joint Surg Am.

[56] Schwab TR, Stillhard PF, Schibli S (2018). Radial nerve palsy in humeral shaft fractures with internal fixation: analysis of management and outcome. Eur J Trauma Emerg Surg.

[57] Bodner G, Huber B, Schwabegger A (1999). Sonographic detection of radial nerve entrapment within a humerus fracture. J Ultrasound Med.

[58] Bodner G, Buchberger W, Schocke M (2001). Radial nerve palsy associated with humeral shaft fracture: evaluation with US–initial experience. Radiology.

[59] Esparza M, Wild JR, Minnock C (2019). Ultrasound evaluation of radial nerve palsy associated with humeral shaft fractures to guide operative versus non-operative treatment. Acta Med Acad.

[60] Cartwright MS, Chloros GD, Walker FO (2007). Diagnostic ultrasound for nerve transection. Muscle Nerve.

