# Supplementary material for: Radial nerve palsy associated with closed humeral shaft fractures: a systematic review of 1758 patients
Source: Arch Orthop Trauma Surg. 2020 Apr 13;141(4):561–8. doi: 10.1007/s00402-020-03446-y (PMC7966639; doi:10.1007/s00402-020-03446-y)
Supplement: Supplementary file 1 — Supplementary file1 (PDF 105 kb) [file 402_2020_3446_MOESM1_ESM.pdf]

**Appendix A** Coleman Methodology Score

| Section | Number or factor | Score |
|---|---|---|
| Part A. Only one score to be given for each section | | |
| 1. Study size: number of patients | <20 | 0 |
| | 20-40 | 4 |
| | 41-60 | 7 |
| | >60 | 10 |
| 2. Mean follow-up | < 12 months | 0 |
| | 12-24 months | 4 |
| | 25-36 months | 7 |
| | > 36 months | 10 |
| 3. Number of interventions per group | Different fixation techniques used, outcome not | 0 |
| (e.g. minimally invasive plate | reported separately | 7 |
| osteosynthesis or non-operative | Different fixation techniques used, outcome reported | 10 |
| treatment) | separately | |
| | Single fixation technique used | |
| 4. Accuracy of diagnosis (location and/or type) | Location and type of fracture described | 5 |
| | Location or type of fracture described | 3 |
| | None described | 0 |
| 5. Type of study | Retrospective cohort | 0 |
| | Prospective cohort study | 10 |
| | Randomized controlled trial | 15 |
| 6. Description of treatment given (fracture treatment) | Adequate (technique stated and necessary details of that type of procedure given) | 10 |
| | Fair (technique only stated without elaboration) | 5 |
| | Inadequate, not stated, or unclear | 0 |
| 7. Description of (postoperative) rehabilitation | Described | 5 |
| | Not described | 0 |
| Part B. Scores may be given for each option in each of the 3 sections if applicable | | |
| Outcome criteria | Outcome measures clearly defined | 2 |
| | Timing of outcome assessment clearly stated | 2 |
| | Use of outcome criteria that has reported reliability | 3 |
| | Outcome with good sensitivity | 3 |
| Procedure for assessing outcomes | Participants actively recruited | 5 |
| | Investigator independent of surgeon | 4 |
| | Written assessment | 3 |
| | Completion of assessment by patients themselves with minimal investigator assistance | 3 |
| Description of subject selection process | Selection criteria reported and unbiased | 5 |
| | Recruitment rate reported > 90% | 5 |
| | Recruitment rate reported = 90% | 0 |
| Total score | | 100 |
