# Supplementary material for: Radial nerve palsy associated with closed humeral shaft fractures: a systematic review of 1758 patients
Source: Arch Orthop Trauma Surg. 2020 Apr 13;141(4):561–8. doi: 10.1007/s00402-020-03446-y (PMC7966639; doi:10.1007/s00402-020-03446-y)
Supplement: Supplementary file 2 — Supplementary file2 (PDF 96 kb) [file 402_2020_3446_MOESM2_ESM.pdf]

**Appendix B** Extracted Data	
Data type	
Study characteristics	Authors last names, journal and year of publication.
Study design	Randomized controlled trial, retrospective cohort study, prospective cohort study, case series
Patient characteristics	Mean age, sex, length of follow up
Type of fracture	Spiral, oblique, transverse, comminuted, segmental
Type of fracture AO-classification	12A1, 12A2, 12A3, 12B1, 12B2, 12B3, 12C1, 12C2, 12C3
Location of fracture	Proximal, middle, distal
Type of intervention	Conservative treatment, operative treatment
Type of operative treatment	Nailing, conventional plating, minimally invasive plate osteosynthesis
Exploration radial nerve	Yes/no
Type of approach	Anterolateral (i.e. minimally invasive anterior, open anterolateral, open extended deltopectoral)<br>Posterior (i.e. open posterior, minimally invasive posterior)<br>Lateral (i.e. lateral open)
Primary radial nerve palsy	Radial nerve palsy occurring at the onset of the fracture
Secondary radial nerve palsy	Radial nerve palsy occurring due to treatment or healing process.
Recovery radial nerve palsy	Recovery of primary or secondary radial nerve palsy without the need of a second intervention
Time to recovery radial nerve palsy	Time to recovery of primary or secondary RNP in weeks
