# Supplementary material for: Radial nerve palsy associated with closed humeral shaft fractures: a systematic review of 1758 patients
Source: Arch Orthop Trauma Surg. 2020 Apr 13;141(4):561–8. doi: 10.1007/s00402-020-03446-y (PMC7966639; doi:10.1007/s00402-020-03446-y)
Supplement: Supplementary file 3 — Supplementary file3 (PDF 124 kb) [file 402_2020_3446_MOESM3_ESM.pdf]

Appendix C Study characteristics

Authors	Year	Closed fractures	Male	Mean age	Follow-up mean (months)	Total radial nerve palsy	Primary radial nerve palsy (recovered)	Secondary radial nerve palsy (recovered)	Intervention(s)
An et al.[30]	2012	34	23	37	22	0	excluded	0	MIPO vs. IMN
An et al.[48]	2010	33	21	37	29	5	excluded	5 (5)	MIPO vs. Plating
Apivatthakakul et al.[31]	2009	20	*	*	14	0	excluded	0	MIPO
Arora et al. [41]	2011	60	*	*	3	3	excluded	3 (*)	Plating
Balam et al. [8]	2014	37	33	28	18	2	excluded	2 (2)	MIPO
Belayneh et al. [9]	2019	98	46	49	7	8	8 (8)	0	Non-operative treatment vs. Plating
Brunner et al. [32]	2012	14	7	64	25	0	excluded	0	MIPO
Chao et al. [33]	2005	92	53	51	71	9	6 (6)	3 (3)	Plating vs. Nailing
Concha et al. [34]	2010	20	*	*	12	0	0	0	MIPO
Dielwart et al. [10]	2017	71	41	38	4	15	15 (13)	0	Non-operative vs. Plating or IMN
Duygun et al. [11]	2017	24	12	42	24	1	0	1 (1)	IMN
Ebrahimpour et al [12]	2015	41	34	39	12	4	3 (3)	1 (1)	Nailing
Ekholm et al. [47]	2008	27	9	51	76	6	6 (6)	0	Non-operative, Plating, IMN
Fan et al. [13]	2015	60	37	39	12	3	excluded	3 (3)	Plating vs. IMN
Galluci et al. [14]	2015	20	12	38	22	1	0	1 (1)	MIPO
Habernek et al. [49]	1992	10	7	*	3	0	0	0	IMN
Han et al. [15]	2017	96	56	43	31	2	excluded	2 (2)	IMN vs. IMN with cerclage cables
Jawa et al. [35]	2006	40	20	45	21	9	6 (5)	4 (2)	Non-operative vs. Plating
Kharbanda et al. [16]	2017	18	12	44	26	2	2 (2)	0	Plating
Ko et al. [17]	2017	50	34	56	19	3	Excluded	3 (3)	MIPO
Lau et al. [36]	2007	17	7	65	14	3	excluded	3 (3)	MIPO

Lee et al. [18]	2018	18	10	35	12	4	4 (4)	0	Plating
Li et al. [42]	2011	45	32	38	12	3	excluded	3 (3)	Plating vs. IMN
Liebergall et al. [37]	1997	19	*	*	36	3	3 (2)	0	IMN
Lin et al. [38]	2003	16	8	40	22	2	2 (2)	0	IMN
Livani et al. [43]	2004	11	9	41	27	0	0	0	MIPO
Malhan et al. [44]	2012	42	28	34	25	1	excluded	1 (0)	MIPO
Matsunaga et al. [19]	2017	110	73	39	12	2	excluded	2 (2)	Non-operative vs. MIPO
Pal et al. [20]	2015	49	26	40	41	6	6 (5)	0	Non-operative
Pehlivan et al. [45]	2002	21	17	25	9	0	0	0	Non-operative
Salvador et al. [21]	2018	60	18	67	11	2	excluded	2 (2)	IMN with cerclage cables
Seo et al. [22]	2019	60	40	44	12	13	9 (9)	4 (4)	Plating
Shen et al. [27]	2013	43	28	40	26	1	excluded	1 (1)	MIPO
Singh et al. [23]	2014	212	148	37	12	25	18 (18)	7 (7)	Plating
Spagnolo et al. [39]	2010	16	10	54	7	0	0	0	MIPO
Wang et al [24]	2018	46	14	71	17	0	Excluded	0	Plating
Yang et al. [40]	2012	19	15	37	17	1	0	1 (1)	Plating
Yin et al. [25]	2014	56	30	38	16	3	excluded	3 (3)	Plating
Zhiquan et al. [46]	2007	13	9	38	13	0	excluded	0	MIPO
Zogaib et al. [26]	2014	20	15	35	52	0	0	0	MIPO

* Unknown value

MIPO = Minimally invasive plate osteosynthesis

IMN = Intramedullary nailing
